# *Raoultella* Bacteremia Presenting as an Acute Self-Limited Illness in an Obstetric Patient

**DOI:** 10.1155/2020/5281792

**Published:** 2020-02-05

**Authors:** Justin Choi, Iqra Sheikh, Melissa Carr, Robert Shapiro, Karen Fluet

**Affiliations:** Department of Obstetrics & Gynecology, West Virginia University School of Medicine, Morgantown, WV. 26506, USA

## Abstract

A 20 year-old female at 27-week gestation was admitted for threatened preterm delivery. Following an initially unremarkable hospital course for 12 days, the patient developed fever, chills, generalized malaise, abdominal pain, and diffuse myalgias on day 13 of hospitalization. *Raoultella* species was isolated from blood cultures on day 16 of hospitalization. The patient's condition improved within 24 hours of symptom onset, prior to antibiotic initiation, and a premature, viable male infant at 29 weeks and 6 days of gestation was delivered via caesarean section four days later due to breech presentation in the setting of preterm labor. Here, we present the first case of a *Raoultella* species infection in a gravid female reported in the literature.

## 1. Introduction

*Raoultella* species belong to the family Enterobacteriaceae and are known to inhabit water, soil, and plants. In humans, they have generally been considered harmless organisms of low virulence that occasionally colonize the gastrointestinal tract and upper respiratory tract, but rarely cause symptoms or infection even in oncologic or immunocompromised patients [1].

*Raoultella* genus is comprised of gram-negative, nonmotile, and encapsulated rods. They are best known for causing scombroid poisoning via the conversion of histidine to histamine. This results in the classic symptoms of cutaneous flushing, urticarial rash, nausea, vomiting, tachycardia, and hypotension. The two most common pathogens, *Raoultella planticola* and *Raoultella ornithinolytica*, have been shown to be capable of causing a wide range of infections in its hosts, with documented clinical manifestations including pneumonia, cholecystitis, pancreatitis, bacteremia, cystitis, and cellulitis [2]. Several cases resulting in death have also been attributed to *Raoultella*. Here, we present the first case of a *Raoultella* species infection in a gravid female reported in the literature.

## 2. Case Presentation

A 20-year-old G2P0100 female at 27-week gestation, with a past medical history significant for hypothyroidism and preterm delivery of a 20-week twin gestation, presented with threatened preterm delivery in the setting of known cervical shortening and new-onset uterine contractions. Cervical examination showed a 1 cm dilated cervix at 80% effacement and a fetal station of -2. Given ongoing contractions, the patient was admitted to the antepartum service. Tocolytics, corticosteroids, and magnesium for fetal neuroprotection were provided. Throughout her admission, fetal testing remained reassuring. Gestational diabetes was diagnosed during routine screening.

Due to continued intermittent contractions, progressive cervical shortening, and previous history of preterm labor, the patient remained in inpatient monitoring. Thirteen days following her initial presentation, the patient reported feeling general malaise as well as a presyncopal episode. The patient was placed on a fetal heart rate monitor revealing both maternal and fetal tachycardia which was unresponsive to an intravenous fluid bolus. Several hours later, she developed fevers, chills, and diffuse myalgias. At that point in time, she was febrile to 38.4°C. Marked tachycardia at 146 beats per minute and tachypnea and fetal tachycardia of 190 beats per minute were also noted. Physical exam was only significant for an infiltrated intravenous catheter in place for magnesium sulfate and fluid boluses as needed. Laboratory evaluation was significant for leukocytosis of 17.9 with a left shift and an elevated C-reactive protein of 37.2. A chest X-ray, urinalysis, and respiratory virus panel yielded no abnormalities.

Shortly following the onset of febrile illness, the patient reported a sense of impending doom. New-onset right lower quadrant abdominal pain was identified on physical examination, sparking concern for appendicitis. An abdominal ultrasound was nondiagnostic; magnetic resonance imaging was negative for acute appendicitis. A diagnostic amniocentesis confirmed the absence of chorioamnionitis, showing no organisms on gram stain or culture.

**Figure 1:**
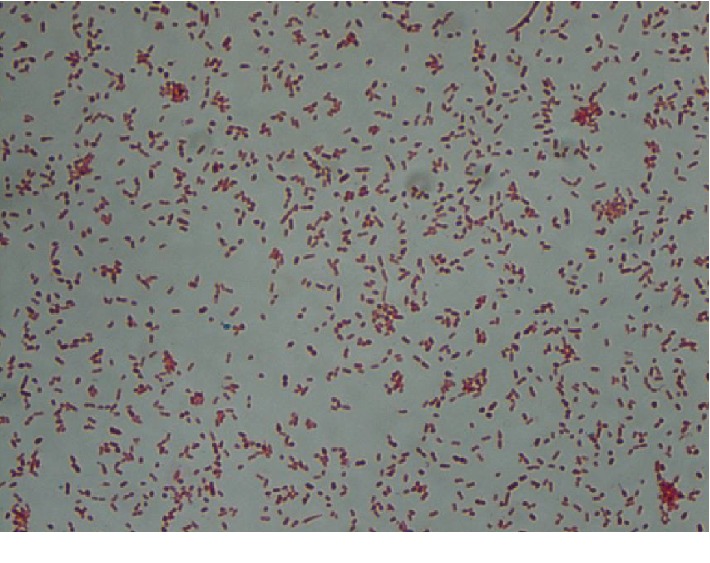
Gram-negative bacteria stain red, which is attributed to a thinner peptidoglycan wall, which does not retain the crystal violet during the decoloring process.

Initial gram staining on blood cultures revealed a gram-negative bacteremia (Figure 1). Empiric treatment with vancomycin and piperacillin-tazobactam was initiated. By that time however, less than 24 hours from initial symptom onset, all symptoms had resolved. The white blood cell count normalized, and the patient remained afebrile throughout the duration of her hospitalization.

Three days later, ampicillin/amoxicillin-resistant *Raoultella* was speciated from the blood cultures. Antibiotics were deescalated to ceftriaxone for seven days, with a total of ten days of antimicrobial therapy. Symptoms never returned, and the patient underwent a preterm delivery of a viable infant with weighing 1.695 kilograms via low transverse caesarean section four days following the acute illness. Placental pathology showed no acute inflammation as well as chorionic villi showing maturation consistent with gestational age.

## 3. Discussion

*Raoultella* is becoming a more clinically relevant organism. While historically an obscure pathogen, *Raoultella* infections are becoming more frequently reported, with twelve recorded cases in April of 2014 rising to over thirty cases by late 2017 [3^,^^4^] and reports of antimicrobial-resistant strains are growing [2]. This genus is often naturally resistant to aminopenicillins and can acquire carbapenemase and extended-spectrum beta-lactamase genes via plasmid acquisition [1].

Several risk factors for *Raoultella* infection have previously been identified, including enteral feeding tubes, prolonged hospitalizations, immunosuppression, long-term antibiotics, diabetes, and catheters, some of which were present in our patient [1]. First, the patient in this report had been hospitalized for almost two weeks, increasing the risk for contact with nosocomial infection via contaminated surfaces or instruments. The patient also had a peripheral IV catheter, providing a possible nidus. Furthermore, she carried the diagnosis of gestational diabetes mellitus (GDM). While isolated GDM has not been shown to have the same immunosuppressive effects as longstanding hyperglycemia associated with uncontrolled diabetes, it has been found to heighten systemic inflammation and intensify the body's response to proinflammatory stimuli such as infection [5]. The patient was also pregnant which creates a unique environment for infection. While pregnancy is not an overtly immunocompromised state, there are elements of maternal immunosuppression in order to prevent the immune system from attacking the semiallogenic fetus [6]. For example, regulatory T-cells modulate the immune system in pregnancy to prevent miscarriage and preeclampsia; complement regulatory proteins such as decay accelerating factor, CD55, and membrane cofactor protein on trophoblastic cells protect the gestational tissue from humoral immunity, and exosomes secreted by syncytiotrophoblasts display an immunosuppressive effect in the mother [7–9]. All of these mechanisms may have contributed to the peculiar presentation of *Raoultella* infection in this patient.

Finally, the patient's preterm delivery, while likely multifactorial, was almost certainly influenced by her infection. Both systemic and genital tract infections have been shown to be associated with preterm birth in multiple large epidemiologic studies [10]. The patient's *Raoultella* bacteremia in this case almost certainly put her at an increased risk of preterm labor.

## 4. Conclusion

Frank infection with *Raoultella* species is remarkably rare, with less than 35 reported cases worldwide as of 2017. The majority of the reported cases occurred in older male patients with known risk factors such as immunosuppression, malignancy, trauma, or invasive procedures [1]. There are many ways in which *Raoultella* infection can manifest clinically depending on the host's comorbidities, from a fulminant sepsis to a brief self-limited illness. Because of its ubiquity in the environment, its colonization of the human microbiome and its ability to acquire antimicrobial resistance, it has the potential to cause serious illness in pregnancy.
